# Comparison of the shear bond strengths of two different polyetheretherketone (PEEK) framework materials and CAD–CAM veneer materials

**DOI:** 10.1186/s12903-024-04247-0

**Published:** 2024-04-12

**Authors:** Gonca Deste Gökay, Seda Üstün Aladağ

**Affiliations:** Department of Prosthodontics, Faculty of Dentistry, Bursa Uludağ University, Bursa, Turkey

**Keywords:** PEEK, BioHPP, Shear bond strength, CAD-CAM materials

## Abstract

**Background:**

This study evaluated the shear bond strength (SBS) of two different polyetheretherketone (PEEK) and CAD-CAM materials after aging.

**Methods:**

A total of 42 frameworks were designed and milled from 2 different PEEK discs (Copra Peek, P and BioHPP, B). P and B frameworks were divided into 3 subgroups (*n = *7). 14 slices were prepared each from feldspathic ceramic (Vitablocs Mark II, VM), hybrid nanoceramic (Cerasmart, CS), and polymer-infiltrated ceramic (Vita Enamic, VE) blocks. After surface preparations, the slices were cemented to P and B surfaces. The samples were subjected to thermal aging (5000 cycles). SBS of all the samples was measured. Fractured surfaces were examined by SEM/EDX analysis. The Shapiro–Wilk, Two-way Robust ANOVA and Bonferroni correction tests were used to analyze the data (a = .05).

**Results:**

Frameworks, ceramics, and frameworks x ceramics had significant differences (*p < *0.05). The highest SBS value was seen in B-VM (*p < *0.05). VM offered the highest SBS with both P and B. The differences between P-VM, P-CS, P-VE and B-CS and B-VE were insignificant (*p >* 0.05). According to EDX analysis, ytterbium and fluorine was seen in B content, unlike P. While VM and CS contained fluorine, barium, and aluminum; sodium and aluminum were observed in the VE structure.

**Conclusion:**

Bonding of P and B with VM offers higher SBS. VM, CS and VE did not make any difference in SBS for P, however VM showed a significant difference for B.

## Background

Polyetheretherketone (PEEK) is a high-performance, semicrystalline, thermoplastic polymer from the polyaryletherketone (PAEK) family [1, 2]. PEEK is a widely used material in dentistry because it is noncorrosive, chemically inactive, nonallergenic, and a polishable, low-molecular-weight polymer that resists plaque formation, high temperatures, water absorption and wear [2–5]. In addition, the elasticity modulus of PEEK (4 GPa), which is much lower than that of titanium (102–110 GPa), is similar to that of bone, enamel and dentin, paving the way for its use in prosthetic applications, such as temporary abutments, implant-supported bar prostheses, and dental implants [1, 6–8]. PEEK has become an alternative to traditional cobalt–chrome frameworks for removing partial dentures because of its ability to eliminate metallic taste and avoid causing allergic reactions in patients with metal allergies; in addition, the material has acceptable dimensional stability and tensile properties close to those of enamel and dentin [7, 9–11]. In implant or tooth-supported prostheses, the choice of framework material is an important step for effectively balancing stress during chewing to eliminate many complications, such as screw loosening, bone loss around the implant, and prosthesis fractures [2, 12, 13]. Owing to its advanced mechanical properties and low elastic modulus, PEEK acts as a stress breaker and distributes forces on restorations [14, 15]. Reportedly, PEEK frameworks produced using computer-aided design (CAD) – computer-aided manufacturing (CAM) are more resistant to deformation and fracture than those produced using pressing technology [16].

In recent years, efforts have been made to improve the mechanical and biological properties of PEEK materials by adding pigments, titanium dioxide and ceramic-containing fillers to their structures [2, 17]. BioHPP is a bioactive, semicrystalline high-performance polymer obtained by adding 20% titanium dioxide to PEEK [1, 6]. The 0.3–0.5-micron-sized ceramic particles enable BioHPP to offer advanced mechanical properties with a homogeneous structure [18, 19]. BioHPP, which has an elastic modulus similar to that of PEEK, might be an important alternative for patients with parafunctional habits, especially in implantology, because of its biological compatibility, satisfactory flexural strength and shock absorber properties [18–20]. Notably, PEEK can be used as a framework material for periodontal protection of support teeth in patients with posterior edentulous ridges and in patients where classical metal frameworks are not preferred due to color and allergic reactions [21].

In addition to its advantages, PEEK has disadvantages that restrict its use, especially in aesthetic areas. PEEK has an opaque grayish-white color and low translucency. For this reason, glass–ceramic-based materials and resin composites containing dimethacrylate (DMA) and methyl methacrylate (MMA) should be applied for veneering [4, 5, 8, 22–24]. PEEK is an inert, hydrophobic polymer with a low surface energy and resistance to different surface treatments [4, 20–22, 24]. This situation has led to the necessity of increasing the bond strength between the PEEK framework material and the veneer suprastructure material. In these studies, PEEK surfaces were modified with various surface conditioning agents, and bonding agents with different contents were applied to the bonding area [1, 4, 10, 22, 23, 25–27]. Reportedly, applying a bonding agent containing pentaerythritol triacrylate (PETIA), DMA and MMA after sandblasting the PEEK surface with aluminum oxide results in increased bond strength [28–30]. Researchers generally focus on the bonding of composite veneers to PEEK framework materials. Studies in which PEEK or modified PEEK framework materials are veneered with suprastructures produced from veneer ceramics are rare [5, 23, 31]. In one study, lithium disilicate ceramic samples were cemented to frameworks made of PEEK and zirconia, and the shear bond strength was examined; further studies comparing other ceramics with lithium disilicate are needed [5]. In another study, lithium disilicate ceramic and indirect laboratory composites were used to veneer two different polymer frameworks from the PAEK family, and it was reported that the polymer type and veneering material affected the shear bond strength [23]. In a clinical case, PEEK was preferred as the framework material for an implant-supported mandibular prosthesis for a completely edentulous patient with an atrophic maxilla and mandible, and lithium disilicate crowns were cemented on the framework [31]. In agreement with these studies, an in vitro study was planned in which current CAD–CAM ceramics, such as feldspathic ceramics, hybrid nanoceramics and polymer-infiltrated ceramics, were preferred as suprastructure materials for comparison in terms of bond strength.

The purpose of this in vitro study was to evaluate the influences of different CAD-CAM veneer materials on the shear bond strengths of two different PEEK framework polymer materials after artificial aging with thermocycling. The null hypothesis was that framework polymer materials and CAD–CAM veneer materials would not change the shear bond strength.

## Methods

In this study, a power analysis was performed using GPower 3.1.2 to determine the sample size. With a 95% confidence level (1-α), 95% test power (1-β) and f = 0.488 effect size, the total number of samples to be included in the study was found to be 60, with 57/6 ≅ 10 samples in each group. When the test power was 80%, a total of 36 samples with 6 in each group should be prepared [22]. Therefore, considering the potential losses, the total sample size was determined to be 42 (7 samples for each group).

The types, compositions and manufacturer information of the framework polymer materials, veneer materials and adhesive agents used are listed in Table 1.

**Table 1:** Description of the polymers, ceramics and adhesive agents used in the study

Brand	Type	Composition	Manufacturer
**Copra Peek**	high performance polymer, polyetheretherketone (PEEK)	PEEK (%100)	White Peaks, Essen, Germany
**breCAM.BioHPP blank**	bioactive high performance polymer, ceramic-reinforced PEEK (Bio-HPP)	Partially crystalline PEEK with 20 wt% ceramic fillers	Bredent, Senden, Germany
**Vitablocs Mark II**	feldspathic ceramic	Silicon dioxide 56–64%, aluminium oxide 20–23%, sodium oxide 9–11%, potassium oxide 6–8%, calcium oxide 0.3–0.6%, titanium oxide 0.0–0.1%	Vita Zahnfabrik, Bad Saöckingen, Germany
**Cerasmart**	hybrid nanoceramic	Resin composite material (BisMEPP, UDMA, DMA) with 71 wt% silica and barium glass nanoparticles	GC Europe, Leuven, Belgium
**Vita Enamic**	polymer-infiltrated ceramic network	Ceramic: Silicon dioxide 58–63%, aluminium oxide 20–23%, sodium oxide 9–11%, potassium oxide 4–6%, boron trioxide 0.5–2%, zirconia and calcium oxide. Polymer (25%): UDMA, TEGDMA	Vita Zahnfabrik, Bad Saöckingen, Germany
**Vita Adiva Cera-Etch**	hydrofluoric acid gel	5% hydrofluoric acid	Vita Zahnfabrik, Bad Saöckingen, Germany
**Vita Adiva C-Prime**	ceramic primer	Solution of methacrylsilanes in ethanol	Vita Zahnfabrik, Bad Saöckingen, Germany
**Variolink Esthetic DC**	dual curing resin-based dental luting material	Monomer matrix: UDMA and methacrylate monomers Inorganic fillers: ytterbium trifluoride and spheroid mixed oxide. Initiators, stabilizers and pigments are additional ingredients The particle size is 0.04–0.2 μm. The total volume of inorganic fillers is approx. 38%	Ivoclar Vivadent, Schaan, Liechtenstein
**Visio.link**	bonding agent, primer	MMA, DMA, PETIA, 2-propenoic acid, activators, stabilizers	Bredent, Senden, Germany

*Bis-GMA* Bisphenol A diglycidil methacrylate, *UDMA* Urethanedimethacrylate, *TEGDMA* Triethyleneglycolimethacrylate, *BisMEPP* 2,2-bis (4 methyacryloxypolyethoxyphenyl) propane, *DMA* Dodecyl dimethacrylate, *PETIA* Pentaerythritol triacrylate, *MMA* Methyl methacrylate

### Sample preparation

In this study, 2 × 12 × 14 mm samples were designed from 2 different PEEK prefabricated discs without filler (Copra Peek, White Peaks, Germany) or with a 20% ceramic filler (BioHPP, Bredent, Germany) using a software program (CATIA V5, Dassault Systemes, France). According to the obtained STL files, 21 PEEK and 21 BioHPP samples were milled using CAD–CAM software (Yenasoft, Yenasoft Software, Turkey). Under water cooling, 14 slices were prepared with a 2-mm thickness using a precision cutting device (Microcut, Metkon, Turkey) from CAD–CAM blocks of feldspathic ceramic (Vitablocs Mark II, Vita Zahnfabrik, Germany), polymer-infiltrated ceramic (Vita Enamic, Vita Zahnfabrik, Germany) and hybrid nanoceramic (Cerasmart, GC, Belgium). The polymer and ceramic sample dimensions were measured using a digital caliper (IP54, Yamer, Turkey). To ensure surface standardization, the bonding surfaces of all the samples were finished with waterproof silicon carbide paper (P600C, English Abrasives, England). All the samples were cleaned with distilled water for 5 min and air dried. The PEEK and BioHPP samples were randomly divided into 3 subgroups to bond ceramic slices to their surfaces (*n = *7). The bonding surfaces of the PEEK and BioHPP samples were roughened with 110-μm aluminum oxide (Al_2_O_3_ blasting sand; Renfert, Germany) particles at a 0.2 MPa pressure for a distance of 5 mm for 15 s. A thin layer of primer (Visio.link, Bredent, Germany) was applied to the surfaces in accordance with the manufacturer's instructions, and the samples were polymerized by light activation for 90 s (Valogrand, Ultradent, USA) at a wavelength of 370–400 nm.

After surface preparation of the PEEK and BioHPP samples, the bonding surfaces of the three different CAD–CAM ceramics (*n = *7) were etched with 5% hydrofluoric acid (Vita Adiva Cera-Etch, Vita Zahnfabrik, Germany) for 60 s, rinsed with distilled water and air dried. Then, primers (Vita Adiva C-Prime, Vita Zahnfabrik, Germany) were applied to the ceramic surfaces with an applicator, which were subsequently dried with an air spray after waiting for 60 s. By using dual curing resin-based cement (Variolink Esthetic DC, Ivoclar Vivadent, Liechtenstein), the ceramics were cemented to the PEEK and BioHPP surfaces by applying finger pressure by the same operator. The resin cement was polymerized for 40 s from each side with a light emitting diode source at an intensity of 1000 mW/mm^2^ (VALO Grand, Ultradent, USA).

All cemented polymer–ceramic samples were held in distilled water at room temperature for 24 h for continuous polymerization. The polymerized samples were aged with 5000 thermocycles (Thermocycler THE-1100, SD Mechatronik, Germany) between 5 °C and 55 °C, and they were held in each bath for 30 s. The transfer time was 5 s, and the samples entered clinical use for approximately 6 months [32].

### Shear bond strength test

Each sample was fixed to a universal test device (Model 3343, Instron, USA) to perform the shear bond strength test and was loaded at 0.5 mm/min until fracture. The maximum force (L) was recorded in Newtons (N). The bonding area was measured by using a digital caliper (IP54, Yamer, Turkey). The shear bond strength (MPa) was calculated by using S = L/A, where L is the load at failure (N) and A is the bonding area (mm^2^).

### Scanning Electron Microscopy (SEM)/Energy Dispersive X-ray Spectroscopy (EDX) Analysis

A sample from each group was selected for scanning electron microscopy (SEM)/energy dispersive X-ray spectroscopy (EDX) analysis. The samples were coated with gold–palladium (Au–Pd), and images of the fractured surfaces were obtained at 300 × magnification via a scanning electron microscope (GeminiSEM 300, Zeiss, Germany). The elemental compositions of the polymer and ceramic materials were determined via EDX (XFlash 6–60 detector, Bruker, USA).

### Statistical analysis

Statistical analysis of the data was performed with Jamovi (V2.3.21; The Jamovi Project, Australia) and SPSS (v25.0; IBM, USA) software. The normality of the data distribution was evaluated by using the Shapiro‒Wilk test. Since the data were not normally distributed and the variances were unequal, two-way robust analysis of variance (ANOVA) with the Walrus package was used to compare the data to avoid violations of assumptions. Multiple comparisons were analyzed with Bonferroni correction. A value of < 0.05 was considered to indicate statistical significance at the 95% confidence interval.

## Results

According to the two-way robust ANOVA results (Table 2), framework polymer materials, veneer materials, and framework polymer materials–veneer materials exhibited significant differences (*p < *0.05).

**Table 2:** Two-way Robust analysis of variance results (ANOVA)

		*p*
**Framework polymer material**	4.77	0.029*
**Veneer materials**	13.86	< 0.001**
**Framework polymer material x Veneer materials**	8.65	0.013*

^*^p < 0.05 **p < 0.001

A statistically significant difference was obtained between the median shear bond strength (SBS) values of the veneer materials and framework polymer materials (*p =* 0.029). The median strength of the PEEK (P) was 4.82 MPa, while the median strength of the BioHPP (B) was 7.98 MPa. A statistically significant difference was found between the medians of shear bond strength compared to those of veneer materials, regardless of the framework polymer material (*p < *0.001). The median strength of Vitablocs Mark II (VM) was 5.93 MPa, the median strength of Cerasmart (CS) was 2.60 MPa, and the median strength of Vita Enamic (VE) was 3.54 MPa. While a significant difference was found between VM and CS (*p < *0.001) and between VM and VE (*p =* 0.003), the difference between VE and CS was not significant (*p =* 0.114).

**Fig. 1:**
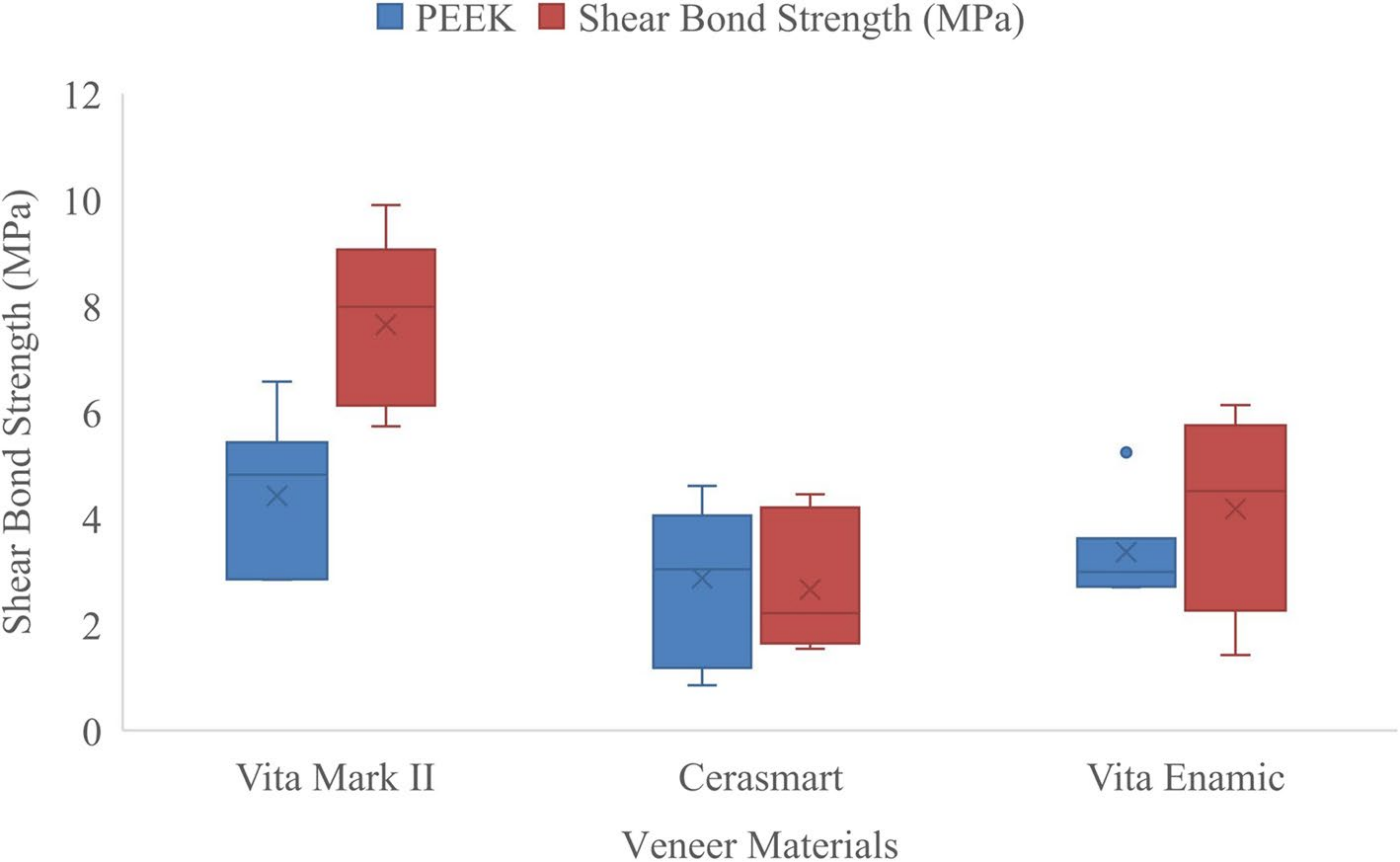
Shear bond strength (MPa) according to framework polymer materials and veneer materials after thermal aging

A statistically significant difference was obtained between the median shear bond strength according to the framework polymer material and veneer materials interactions (*p =* 0.013). The median shear bond strength values (minimum–maximum; MPa) are presented in Table 3 (*p < *0.05). The shear bond strengths of framework polymer materials and veneer materials are presented in Fig. 1 as a box plot.

**Table 3:** Median (minimum–maximum) values and multiple comparisons of shear bond strength (MPa) according to framework polymer materials and veneer materials

Veneer materials	**Framework polymer materials**	
	**PEEK (P)** **median (minimum–maximum) values**	**BioHPP (B)** **median (minimum–maximum) values**
**Vitablocs Mark II (VM)**	4.82 (2.84—6.57) Aa	7.98 (5.73—9.90) Ba
**Cerasmart (CS)**	3.04 (0.85—4.61) Aa	2.21 (1.54—4.45) Ab
**Vita Enamic (VE)**	2.99 (2.70—5.24) Aa	4.51 (1.42—9.90) Ab

Different lowercase letters in the same column and different uppercase letters in the same row indicate statistically significant differences (*p < *0.05)

VM exhibits higher bond strength values with PEEK (4.82 MPa) than CS and VE. Although VE has the lowest bond strength (2.99 MPa) when PEEK is chosen as the framework material, there is no statistically significant difference between P-VM, P-CS and P-VE (*p >* 0.05).

The highest bond strength values among all the groups are observed when the BioHPP framework material is bonded with VM (7.98 MPa), while the lowest bond strength is between B-CS (2.21 MPa). According to multiple comparisons, the differences between the B-CS and B-VE groups are not statistically significant (*p >* 0.05). The interaction effects of B-VM on both B-CS and B-VE are statistically significant (*p < *0.05).

VM has the highest bond strengths with both PEEK (4.82 MPa) and BioHPP (7.98 MPa), and the difference between B-VM and P-VM is statistically significant (*p < *0.05). A higher bond strength can be achieved when CS is bonded with PEEK (3.04 MPa), while VE has a greater bond strength with BioHPP (4.51 MPa). However, these differences for CS and VE are not statistically significant (*p >* 0.05).

**Fig. 2:**
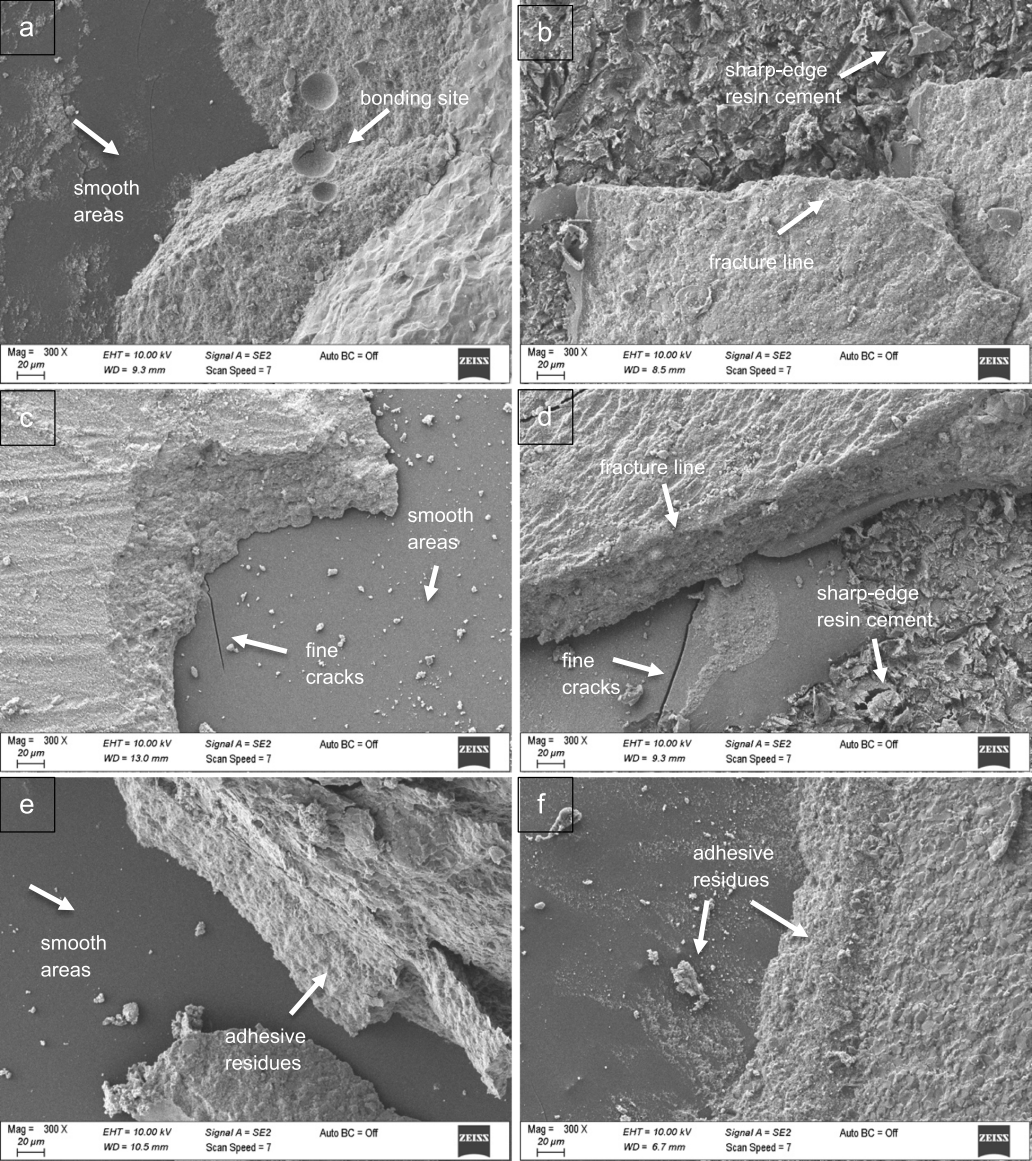
SEM images of fractured surfaces (300 × original magnification). **a** PEEK-Vitablocs Mark II (P-VM). **b** BioHPP-Vitablocs Mark II (B-VM). **c** PEEK-Cerasmart (P-CS), **d** BioHPP-Cerasmart (B-CS). **e** PEEK-Vita Enamic (P-VE), **f** BioHPP-Vita Enamic (B-VE)

SEM images of fractured veneer materials according to the framework polymer material are presented in Fig. 2.

On the surfaces where the veneer materials are debonded from the framework polymer material, BioHPP is rougher than PEEK and contains adhesive residues. Compared to those of BioHPP, the PEEK samples have smoother areas reflecting adhesive bond failures. HF acid provides an adhesive resin bonding area by making the surfaces irregular in all ceramics. Fragmented fractures occur in the ceramics. Sharp-edged resin cement and ceramic residues are prominent on the B-VM and B-CS surfaces. Fine cracks can be observed in the polymer primer structure on the surfaces of P-CS and B-CS. A porous and ringed bonding site can be observed on the P-VM surface.

**Fig. 3:**
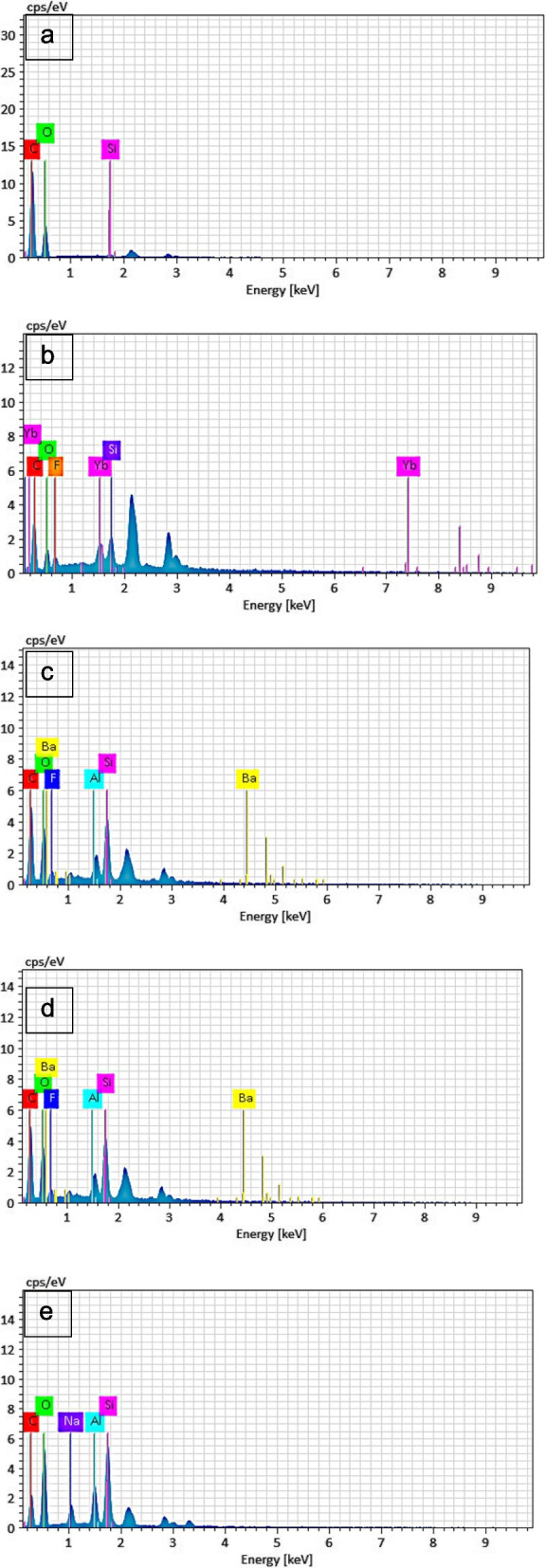
EDX analysis of the framework polymer materials and veneer materials (by weight). **a** PEEK. **b** BioHPP. **c** Vitablocs Mark II. **d** Cerasmart. **e** Vita Enamic

EDX analysis findings of the framework polymer materials and veneer materials are presented in Fig. 3.

PEEK contains approximately 63.97% carbon, 35.10% oxygen and 0.92% silicon by weight. BioHHP contains approximately 47.81% carbon, 16.47% oxygen, 14.56% silicon, 14.35% ytterbium, and 6.81% fluorine by weight. Vitablocs Mark II contains approximately 49.29% carbon, 28.33% oxygen, 14.74% silicon, 4.34% fluorine, 2.28% aluminum, and 1.02% barium by weight. Cerasmart contains approximately 44.32% carbon, 22.33% barium, 13.58% oxygen, 9.87% fluorine, 6.78% silicon, and 3.12% aluminum by weight. Vita Enamic contains approximately 31.56% carbon, 38.47% oxygen, 19.49% silicon, 6% sodium, and 4.47% aluminum by weight.

## Discussion

The results of this in vitro study show that different polymer framework materials and CAD–CAM veneer materials significantly affect the shear bond strength; thus, the null hypothesis is rejected.

PEEK is an inert polymer due to its resonance-stabilized chemical structure and possession of electrons that are not associated with a single atom or covalent bond [5]. In order to improve the mechanical properties and bioactivity of this polymer, special ceramic fillers were added to its structure to obtain BioHPP, a bioactive, thermoplastic high-performance polymer [2, 17, 18]. The low surface energy and resistance of PEEK and BioHPP to different surface treatments and chemicals negatively affect their ability to provide adequate and long-term bond strength [4, 15, 23, 24]. Therefore, it is important to modify the material surface and provide mechanical interlocking and chemical conditioning to establish durable bonds and increase wettability [5, 29, 30]. Depending on their chemical content, adhesive agents can change bond strength and strengthen the bond with a multifactorial effect between the framework material and the resin [15, 25]. Visio.link is the preferred bonding agent in this study. Visio.link, which is used as a primer to bond polymethylmethacrylate- and composite-based artificial teeth and veneers with the framework material, contains MMA, DMA and PETIA [30]. Visio.link activates the material surface on which it is applied by dissolving it. The resulting double carbon bonds polymerize by bonding with the carbon bonds in the structures of the bonding agent and adhesive cement [28]. Researchers have reported that PETIA is particularly effective at modifying the surfaces of PEEK and BioHPP and that Visio.link has a high bond strength with these framework materials [15, 28, 30].

In this study, sandblasted surfaces are treated with aluminum oxide, which has been tested previously, and it is preferred for modifying the PEEK and BioHPP surface [1, 11]. Sandblasting with aluminum oxide roughens the surface by breaking the carbon‒carbon and carbon‒hydrogen bonds in the polymer structure and facilitates the flow of the adhesive agent into the material by increasing the wettability and micromechanical interlocking area [23, 24, 27]. In addition, free radicals released by breaking contribute to chemical bonding by triggering a new chain reaction between resin-containing adhesives and polymers [8, 27]. When the median values are considered, BioHPP (7.98 MPa) exhibits significantly greater SBS values than PEEK (4.82 MPa). The bond strength is a multifactorial parameter that depends on the PEEK filler content, crystallinity, free surface energy, surface roughness, contact angle, and the chemistry of the material to which the surface treatment is applied [1, 11, 27]. Additionally, since BioHPP is developed for use as a PEEK material because of its advanced mechanical properties, it should increase the SBS.

In this study, the bonding surfaces of suprastructure materials are prepared by etching with 5% hydrofluoric acid (HF). VM exhibits the highest bond strength with BioHPP (7.98 MPa), but when cemented to the PEEK surface, there is no significant difference between the suprastructure materials (*p >* 0.05). Moreover, the differences between the CS and VE are not significant, regardless of the difference in framework material (*p >* 0.05). HF acid reacts with the silica contained in silica-based ceramics, such as feldspathic ceramics, to form hexafluorosilicates. Many scholars have reported that conditioning the surfaces of etchable ceramics with HF acid increases the surface roughness and wettability to support bond strength while releasing hydroxyl groups that provide bonding with chemical adhesive agents, such as silane [33, 34]. Therefore, the fact that VM is more affected by HF surface treatment because it is a silica-based feldspathic ceramic may result in high bond strength values.

According to some researchers, VE, a polymer-infiltrated hybrid ceramic with a feldspathic ceramic component, is among the ceramics that can be etched with HF, and HF is a preferable surface treatment for VE [35, 36]. In contrast, one author reported that the etched VE surface has a microporous structure with a partially exposed ceramic matrix and a persistent, undissolved polymer matrix [37]. In another study, scholars reported that when the VE surface is etched with 5% HF, a sufficiently hydrophilic surface is not formed [38]. The VE surface has a hydrophobic surface due to the resin network, which becomes dominant and permanent when acidified [39]. In fact, the resin contents of adhesive cementation materials and the exposed resin network at the VE content can be considered to establish a durable bond. However, in the entire structure, there is a small amount of organic resin matrix structure that contributes to the copolymerization of free monomers and adhesive agents [37, 40]. Furthermore, since the resin matrix structure does not provide sufficient reactive bonds, reliable bonding does not occur [41, 42]. In our study, the fact that VE exhibits lower bond strength than VM can be attributed to these findings.

Blocks with dispersed fillers are obtained by blending the fillers into a matrix structure containing UDMA and TEGDMA under high temperatures and pressures [40]. With this type of polymerization specific to CAD–CAM materials, the degree of conversion reaches 95–96%. As a result, it can be argued that the amount of free monomers and the possibility of interaction with resin cements in the CAD–CAM material structure are reduced [36, 40, 43, 44]. In one study, it has been reported that approximately half of the glass in the CS structure is barium glass. Barium does not react with HF, and a large silicon (Si) containing a surface layer that reacts with coupling agents, such as silane, is needed to obtain a high bond strength. HF acid is insufficiently effective for changing the surface properties of CS [45]. In another study, micromechanical and chemical surface treatments were applied to eight different CAD–CAM resin composite blocks. The reason composite blocks with dispersed fillers are not sufficiently affected by HF acid may be because the filler content does not consist of pure silicon dioxide and because the crystalline mineral component remains outside the effect of HF [36]. The results of these studies may help explain why CS has low bond strengths.

According to the ISO 10477 standard, the SBS value at the interface formed by resin-based materials and framework materials is at least 5 MPa [46]. Conversely, researchers have shown that it is clinically acceptable for resin-based materials to have a minimum SBS value in the range of 10–12 MPa under intraoral conditions [47, 48]. Although not all of the suprastructure materials used in this study are resin based, the SBS values between the framework and suprastructure materials are not within the clinically acceptable threshold range. Only the bonding between VM and BioHPP is above the minimum SBS value. The facts that the suprastructure materials tested are not veneer resins but rather CAD–CAM materials with ceramics and that the bond strength is material dependent likely impact the results. In addition, the application of HF acid, which is a classical method for preparing the surfaces of superstructure ceramics, is preferred. Higher bond strength values can be obtained when subjected to different surface treatments.

In this study, a resin-based dual-cure resin cement is used for the cementation of suprastructure materials. In a study, the tensile bond strength (TBS) of PEEK with methyl methacrylate (MMA) and composite-based resin cements is evaluated, and higher bond strength values are obtained with MMA-based resin cements [25]. During the thermal aging process, water leaks into the cement and PEEK interface and disrupts bonding via hydrolysis. As a result of clinical use reflecting 2 years of intraoral conditions in composite-based cement groups, the TBSs are approximately zero. Separation can be observed between some samples during the thermal aging process. Based on this study, not using MMA-based adhesive resin may have an impact on the inability to obtain clinically acceptable bond strengths.

The bond strength between PEEK and BioHPP framework polymers and veneer materials produced using CAD-CAM blocks with different contents guides the clinical indication and material selection. According to the results of this in vitro study, the bonding between BioHPP and Vitablocs Mark II appears to be stronger. BioHPP may be preferred to PEEK due to its higher bond strength. However, since the bonding between BioHPP and feldspathic ceramic is above the minimum SBS value according to ISO10477 standards, the bonding between CAD-CAM veneer materials and PEEK materials needs to be improved.

Even if thermal aging is applied to the materials, the limitations of the study include the inability to simulate the intraoral conditions exactly and the lack of occlusal forces and pH changes, and the fact that the samples were kept in distilled water rather than saliva until testing. There is a need to plan additional studies in which the surfaces of PEEK materials and CAD–CAM veneer suprastructure ceramics are conditioned by different surface treatments, such as cold atmospheric plasma, sandblasting and lasers, and where adhesive resins with different contents are used.

## Conclusions

Based on the findings of this in vitro study, the following conclusions are drawn:

1. Although VM has higher SBS values with PEEK than with CS or VE, there are no differences between VM, CS and VE for the PEEK framework material.
2. The highest SBS values can be observed between BioHPP and VM. For the BioHPP framework material, VM is significantly different from both CS and VE, while there is no difference between CS and VE.
3. The SBSs between BioHPP and VM are above the minimum SBS threshold according to ISO10477. The SBS values between all framework and suprastructure materials are below the recommended clinically acceptable value range.
4. According to EDX analysis, ytterbium and fluorine was seen in B content, unlike P. While VM and CS contained fluorine, barium, and aluminum; sodium and aluminum were observed in the VE structure.

## Data Availability

The datasets used and/or analysed during the current study are available from the corresponding author on reasonable request due to privacy reasons and large data size.

## Abbreviations

PEEK: Polyetheretherketone
PAEK: Polyaryletherketone
Bio-HPP: Bioactive high performance polymer
CAD: Computer-aided design
CAM: Computer-aided manufacturing
bis-GMA: Bisphenol A diglycidil methacrylate
UDMA: Urethanedimethacrylate
TEGDMA: Triethyleneglycolimethacrylate
bisMEPP: 2,2-Bis (4 methyacryloxypolyethoxyphenyl) propane
DMA: Dodecyl dimethacrylate
PETIA: Pentaerythritol triacrylate
MMA: Methyl methacrylate
